# Implicit neural representations in light microscopy

**DOI:** 10.1364/BOE.515517

**Published:** 2024-03-06

**Authors:** Sophie Louise Hauser, Johanna Brosig, Bhargavi Murthy, Alessio Attardo, Andreas M. Kist

**Affiliations:** 1 Department Artificial Intelligence in Biomedical Engineering, Friedrich-Alexander-Universität Erlangen-Nürnberg (FAU), Germany; 2 Charité – Universitätsmedizin Berlin, Germany; 3 Leibniz Institute for Neurobiology, Germany

## Abstract

Three-dimensional stacks acquired with confocal or two-photon microscopy are crucial for studying neuroanatomy. However, high-resolution image stacks acquired at multiple depths are time-consuming and susceptible to photobleaching. In vivo microscopy is further prone to motion artifacts. In this work, we suggest that deep neural networks with sine activation functions encoding implicit neural representations (SIRENs) are suitable for predicting intermediate planes and correcting motion artifacts, addressing the aforementioned shortcomings. We show that we can accurately estimate intermediate planes across multiple micrometers and fully automatically and unsupervised estimate a motion-corrected denoised picture. We show that noise statistics can be affected by SIRENs, however, rescued by a downstream denoising neural network, shown exemplarily with the recovery of dendritic spines. We believe that the application of these technologies will facilitate more efficient acquisition and superior post-processing in the future.

## 1. Introduction

Light microscopy is an integral part of neuroscientific research. The visualization of individual neurons and their related sub-anatomy, such as dendritic spines, is essential for studying connectivity, memory, and learning. Dendritic spines are small protrusions found on dendrites, constituting the postsynaptic component of the majority of excitatory synapses in the brain. It has been proposed that dendritic spines act as the fundamental units of neuronal integration in the brain [1] as well as being the seat of information storage [2]. In terms of their structure, dendritic spines are characterized by a rounded head, which is connected to the dendrite by a slender spine neck [3].

Ideally, these imaging studies are performed in awake, behaving animals, to uncover activity-related or time-variant anatomical changes. However, the living organism not only constrains the experimenter in optical setups but also induces motion artifacts that confound the image quality and therefore affect the downstream analysis.

The use of artificial intelligence, especially deep learning methodologies, has seen huge attention in biomedical imaging recently [4,5]. For many applications, deep neural networks largely outperform classical algorithms for computer vision and image reconstruction. Weigert et al. are using a convolutional neural network to alleviate the issue of severe anisotropy in axial versus lateral resolution of 3D fluorescence microscopy images [6]. While other techniques and deconvolution methods addressing this problem suffer from high time consumption or limitations in their ability to reduce anisotropy, their network is able to effectively learn to restore the full isotropic resolution. Wu et al. proposed a deep neural network in fluorescence microscopy, which virtually refocuses a 2D image onto user-defined 3D surfaces within the sample [7]. This approach is able to correct tilt, sample drift, and other aberrations. Their method can potentially replace other scanning and non-scanning methods, which are limited by imaging speed and throughput, phototoxicity, and photobleaching.

However, these solutions have a drawback from the depletion of high-frequency details and are limited in adjacent frame interpolation. Recently, deep neural networks that incorporate Fourier features show superior performance especially in recovering high-frequency details [8,9].

Implicit neural representations aim to parameterize discrete signals as continuous functions within neural networks. These networks are trained to model this continuous function by mapping the domain of the input signal to the target outputs. Sinusoidal representation networks (SIRENs) [9] are capable of representing high-frequency natural signals and their derivatives. This is a fundamental requirement for our work, because of the fine texture of the neural anatomy in the microscopic images we are analyzing. For our tasks, the SIRENs were trained to map the pixel grid of a microscopic image stack to the corresponding pixel values of the image stack. Effectively, our image stacks are a discrete $R^{3}\mapsto R^{1}$ mapping from a 3D coordinate space to a 1D grayscale value. SIRENs are utilized to model this mapping as a continuous function Φ taking the pixel coordinates as inputs and returning the corresponding grayscale values. Simply put, we use SIRENs as an alternative way of representing our image stacks. Therefore, each SIREN implicitly represents exactly one image stack. Modeling fine details with the function Φ is then not limited by the grid resolution anymore but by the network architecture capacity.

Implicit neural representations based on SIRENs were already used successfully by Lei et al. [10] for photon propagation in a monolithic neutrino detector to model individual photon propagation. Since SIRENs are able to learn an underlying functional shape, they are scalable to larger detectors, which is poorly feasible with traditional methods. Furthermore, recent works have illuminated promising advancements in the realm of microscopic data analysis. Wiesner et al. [11] represent living cell shapes in a 3D+time domain as level sets of signed distance functions utilizing SIRENs. This technique allows for the generation of new datasets crucial for training deep learning models for reliable and accurate segmentation. Their models are able to accurately reproduce biological processes like mitosis, cell growth, and branching of cells. Byra et al. [12] leverage SIRENs for the registration of 2D microscopy *in situ* hybridization gene expression images of the marmoset brain. They pairwise register images with similar anatomical structures, where one image contains additional features, which are not contained in the other image. Their method outperformed all other methods with which they have compared themselves in four out of five brain regions. Additionally, their method offers the advantage of extracting microscopy artifacts and therefore reduces their impact on image registration.

In this study, we investigated novel potential applications of SIRENs within the realm of light microscopy. Specifically, we evaluated if SIRENs are suited to predict intermediate planes (interplanes) of microscopic image stacks and thereby increase the spatial resolution along the z-axis. We further hypothesize that implicit neural representations are capable of deciphering consistent, stable data from variant, motion-afflicted data. The key innovations of this research comprise a non-biased and unsupervised approach designed to eliminate motion artifacts in microscopic images. Additionally, both the time requirements and negative side effects associated with data acquisition are potentially reduced, as only every n-th plane needs to be acquired and the intermediate planes are reconstructed by the SIRENs.

## 2. Methods

### 2.1 Two-photon microscopy and data generation

Dendritic spine data was acquired as described in [13] by using a Bruker Ultima IV microscope and a 25x water immersion objective (Olympus XLPlan N 25x/1.00 SVMP). To excite eGFP we used a pulsed infrared laser tuned to 920 nm. To image dendritic spines, we acquired z-stacks (48.18- μ $m^{2}$ single section area, 1- μ m z-step, 5–70 z-steps, zoom 10x, 28.6- to 115.5-mW laser power at the sample) of the dendrite of interest using a resonant scanner at each time point. We acquired each z-plane four times before moving to the next plane.

### 2.2 SIRENs

We are using a multilayer perceptron (MLP) architecture with periodic sine activation functions as our SIREN implementation [9]:

(1) $$\Phi (x)=W_{n}(\phi_{n-1}\circ \phi_{n-2}\circ \ldots \circ \phi_{0})(x)+b_{n}$$

(2) $$\phi_{i}(x_{i})=\sin(\omega_{0}\cdot W_{i}x_{i}+b_{i}).$$

$\phi_{i}:R^{M_{i}}\mapsto R^{N_{i}}$ is the $i^{th}$ layer of the SIREN. Every layer consists of an affine transformation defined by a weight matrix $W_{i}\in R^{N_{i}\times M_{i}}$ and biases $b_{i}\in R^{N_{i}}$ , which are applied on the input $x_{i}\in R^{M_{i}}$ . The frequency tuning is determined by $\omega_{0}$ . On each component of the resulting vector, the sine linearity is applied. Using the above architecture, a continuous function Φ can be obtained, parameterizing the input signal.

Prior to the training of the SIRENs, the microscopic image stacks were normalized with a percentile normalizer as follows:

(3) $$y_{normalized}=\frac{y-P_{2}}{P_{99.9}-P_{2}+\epsilon }$$

where y are the pixel values of the image stack, $P_{2}$ is the second percentile of y , $P_{99.9}$ is the 99.9th percentile of y and $y_{normalized}$ are the normalized pixel values. We use $\epsilon =1\times 10^{-20}$ to ensure, the denominator is always greater than zero. The input data was further preprocessed depending on the task. For the motion correction task, we used four samples of one image stack with an unknown component of motion artifacts. To preprocess these stacks in order to correct the motion artifacts, we investigated two main approaches. The first one was to use all repetitions of the plane samples. Their corresponding grids were stacked in the same way and used as the input data while the grayscale values served as ground truth. For the second approach, we built a new image stack by randomly sampling pixel values from all repetitions. The values of this stack and the corresponding grid were then used as ground truth labels and input data respectively. By learning the implicit representations modeling the underlying signal with SIRENs, uncorrelated motion artifacts can be eliminated. We compared our approach using SIRENs with a state-of-the-art convolutional neural network (CNN) to correct motion artifacts proposed by [14]. This supervised network essentially consists of three convolution layers. The first two layers contain 64 filters and a ReLU activation function, and the last layer has one filter and no activation function. The kernel size of all layers is 5 × 5. We trained the network with stochastic gradient descent with a learning rate of 0.01 and mean-squared error loss similar to [14]. As training data, we used 114 image planes from 3 image stacks and 20 image planes from one image stack as test data. To create ground truth images, we manually selected the repetitions without motion artifacts and averaged them.

To train the SIRENs for interplane prediction, we used the following procedure. First, we systematically selected specific planes to be used as training data. After the SIREN learned the implicit representation of this training image stack, the grayscale values at the pixel coordinates of the omitted planes were predicted by the SIREN. These predicted values were then evaluated by comparing them to the original grayscale values. To progressively challenge the model’s performance, we gradually increased the number of omitted planes with each iteration. In the initial run, every second image plane was utilized for training, while the skipped planes were reconstructed using the SIREN. In the subsequent run, every third plane was employed for training, with the intermediate planes being predicted by the SIREN. This iterative procedure was repeated until only every eighth plane was used for training, while the remaining seven planes in between were excluded and subsequently predicted by the SIREN. A visualization of this procedure is shown in Fig. 3(d). Additionally, we compared our approach of reconstructing interplanes with an alternative method. Therefore we have chosen linear interpolation.

Deep Neural Networks were set up in TensorFlow v2.10 using the high-level Keras package. Our SIREN MLPs contain three hidden layers with 128 units each. Weights were randomly initialized and drawn from a uniform distribution such as $w_{i}∼U(-\sqrt{6/n},\sqrt{6/n})$ , where n is the number of inputs to the hidden layer. The hyperparameter $\omega_{0}$ was set to 32 for the input layer and 34 for the hidden layers. The outermost layer has no activation functions. We optimized the weights using the Adam optimizer with a constant learning rate of 0.001 together with the mean squared error (MSE) loss. For every 3D microscopic image stack, a SIREN was trained for 1500 steps. In each step, the input data (the pixel grid of the image stack) and corresponding labels (the pixel values) were first shuffled and then the network was fit to this data in one epoch with the whole dataset in one batch. The networks were trained on an NVIDIA GeForce RTX 3090 GPU.

We computed the mean squared error, the structural similarity index measure (SSIM, [15]), and the peak-signal-to-noise ratio (PSNR) to evaluate how close our prediction is compared to the respective reference image.

### 2.3 Spine recovery

To improve the spine recovery of the predictions, we use a custom denoising encoder-decoder network inspired by the U-Net architecture introduced by Ronneberger et al. [16]. This network was trained with SIREN representations of 1545 microscopic images from 38 image stacks as training data and the corresponding originally acquired images as ground truth to model the noise statistics of the original microscopy stacks. In this way, the SIREN predicted images are corrected to resemble more closely real microscopic images. Similar to the U-Net [16] we use a filter scaling approach of 64 filters in layer 1, 128 in layer 2, etc. However, we used a filter size and a kernel size of 1 in the output layer. The weights were optimized using the Adam optimizer with a constant learning rate of 0.01 and the MSE loss. The model was trained for 100 epochs with a batch size of 10 images.

We evaluated the spine recovery with the DeepD3 framework, a dedicated tool for the detection of dendritic spines and dendrites [17]. DeepD3 assigns a probability to every pixel for being a spine or dendrite respectively. To fine-tune the spine identification algorithm and count the number of spines in each image plane, we used the DeepD3 graphical user interface (GUI). We utilized a pre-trained DeepD3 model with 32 base filters to count the spines in each slice of a predicted stack and compared it to the number of spines in the original image stack. To evaluate the performance of the denoiser network we computed the mean absolute error and standard deviation of the spines in the SIREN predictions and compared them to the SIREN predictions which were corrected by the denoiser network.

**Fig. 1.**
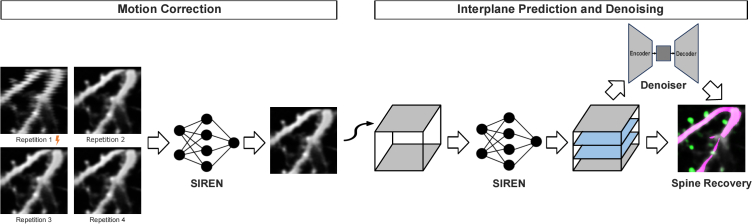
**Processing pipeline**. SIRENs will be used to create a motion-corrected and denoised microscopy stack from multiple repetitions of the same area. Subsequently, interplanes can be predicted with another SIREN model. Input data for the SIRENs are the spatial coordinates (x, y, z) of the image stacks, output values are the corresponding intensity values. SIREN-derived image quality is optionally further processed by a downstream denoiser network to improve spine recovery.

The whole processing pipeline is shown in Fig. 1. In the first part, one image stack is predicted by a SIREN from several repetitions. In this step, motion artifacts are corrected. The resulting stack is then again implicitly represented by a SIREN and an individually chosen number of interplanes can be predicted. These predictions can be optimized in a postprocessing step by a denoiser network. In this way, we are able to create an image stack with corrected motion artifacts and an increased resolution in the z-direction.

## 3. Results

### 3.1 Motion correction

*In vivo* acquisition of microscopic data is prone to motion artifacts. Multiple acquisitions of the same plane and subsequent averaging are common approaches to cope with motion and acquisition noise. However, even through averaging motion artifacts mathematically contribute to the resulting data and may impact downstream data analysis. A supervised approach that manually removes planes with motion artifacts is also cumbersome and in large-scale data analysis not feasible. Therefore, we propose a non-biased and unsupervised method to allow for the elimination of motion artifacts for large amounts of data. We hypothesize that deep neural networks could utilize implicit representations to model the underlying signal and avoid uncorrelated motion artifacts.

**Fig. 2.**
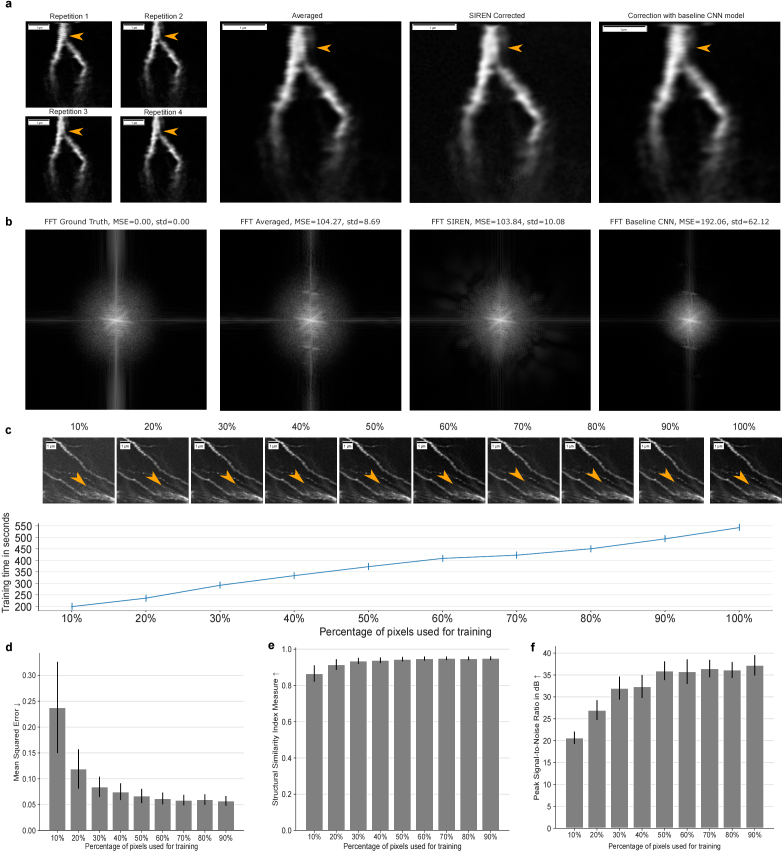
**Quantitative and qualitative evaluation of motion correction performed by SIRENs. a**, Four repetitions of one image plane, where repetition one is affected by a motion artifact. The average of all repetitions, the SIREN prediction of the image plane, and the prediction of the baseline CNN (from left to right). **b**, The Fourier transform of the ground truth, the averaged repetitions, the SIREN corrected image, and the baseline CNN for the example image shown in **a**.**c**, Using only a limited amount of pixels for training. Predictions of one image plane for 10% to 100% used pixels (top). Predictions get noisier with decreasing usage of pixels during training. The training time increases with the number of used pixels in an approximately linear way (bottom). Times were measured for an image stack with 20 planes randomly sampled from all repetitions as ground truth. **d-f**, Using only a limited amount of pixels for training. Evaluation of the MSE (d), SSIM (e), and PSNR(f) of the respective predictions compared to using all pixels during training.

Figure 2(a) shows four repetitions of the same image plane, where the first repetition is affected by a motion artifact. To the right of the repetitions, the result of averaging the repetitions can be seen, next to the SIREN prediction for correcting the motion artifact, and then the result of the correction with the baseline CNN. The motion artifact is still visible in the averaged stack and the stack corrected with the baseline CNN, whereas it is no longer visible in the SIREN-predicted image.

We investigated two main approaches to train the SIRENs in order to correct motion artifacts. For the first approach, we used all four plane samples in the training paradigm as ground truth. For the second approach, we built a new image stack by randomly sampling pixels from the different repetitions and trained the SIRENs with this stack. We evaluated these methods by manually selecting the repetitions without motion artifacts, averaging them, and using this averaged stack as the ground truth. Both methods performed almost equally concerning mean squared error, structural similarity index measure, and peak-signal-to-noise ratio. However, the training in the second approach was substantially faster with a training time of about 9 minutes compared to using all repeated image stacks completely, which took 38 minutes to train. This shows a linear correlation between the amount of training data and the required training time.

To evaluate our predictions and compare these to the results of averaging the repetitions and the predictions of the baseline model, we computed the Fourier spectrum of the images. As ground truth, we used the average of the repetitions without motion artifacts. We compared the Fourier spectra of the different methods to the ground truth by calculating the MSE for every plane. The MSE of the SIREN predictions was slightly lower than for the averaged images with 103.84 ± 10.08 compared to 104.27 ± 8.69. It is important to note, that averaging performs worse from a visual perspective since the motion artifacts can still be seen in the output images. The low MSE can be justified by the fact, that motion artifacts typically impact only parts of the image. The not-impacted parts are nearly equal to the ground truth when averaging the repetitions. Both methods outperformed the baseline CNN, which shows an MSE of 192.06 ± 62.12. The Fourier transform for one example image plane is shown in Fig. 2(b).

Additionally, we evaluated, how well the SIRENs perform with a limited amount of pixels as training data. We systematically varied the relative amount of the total available data to investigate which amount is sufficient to reconstruct accurately the 3D image stack. Figure 2(c) (upper part) shows qualitative results for different pixel percentages. Already with only 10% of the pixels used to train the SIREN, the general structure of the neuroanatomy can be reconstructed. With the increasing usage of the data, the predictions exhibit less noise. Already half of the data provides qualitatively good results with only a small amount of noise, and no severe differences to using the full training data can be visually identified. Using a reduced percentage of data points for training results in shorter training times, which is important for time-critical applications. The training times increase approximately linear with the number of pixels used and therefore the amount of data processed during training, as shown in Fig. 2(c) (lower part). Times were measured for an image stack with 20 planes and the preprocessing approach of creating a randomly sampled stack of the four sample repetitions as training data. This confirms the finding when comparing the two different approaches to training SIRENs to correct motion artifacts. Training time increases linearly with the amount of training data. We additionally quantitatively evaluated how well SIRENs can cope with the reduced data amount relative to SIRENs trained on the full data using the MSE, SSIM, and PSNR metrics. The results are shown in Fig. 2(d)-(f). We observe that using only 60% of the data can already provide predictions that are really close to the results acquired by using the full data. When using even fewer data, the scores of all previously mentioned metrics get worse exponentially.

### 3.2 Interplane prediction

**Fig. 3.**
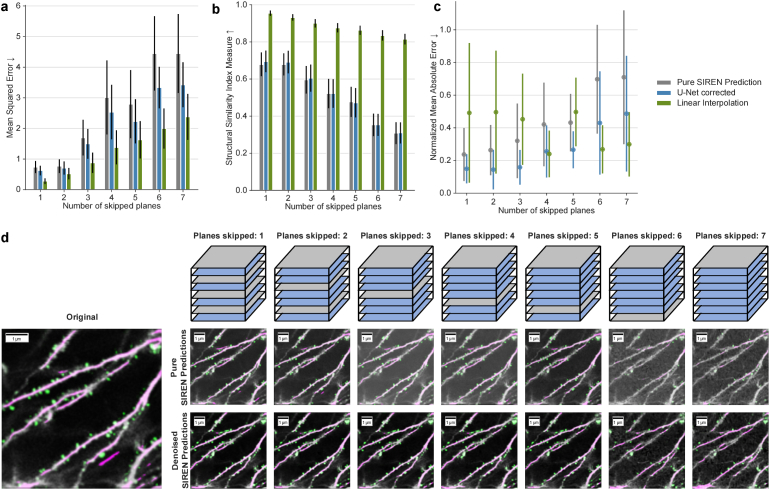
**Qualitative and quantitative evaluation of interplane prediction. a**, Mean squared error between pure SIREN predictions and original images (gray), denoised SIREN predictions and original images (blue), and linear interpolation and original images (green) for different numbers of skipped planes. **b**, Structural similarity index measure between pure SIREN predictions and original images (gray), denoised SIREN predictions and original images (blue) for different numbers of skipped planes, and linear interpolation and original images (green). **c**, Exemplary evaluation of spine recovery for one image stack. The number of spines in each image stack was predicted by a pre-trained DeepD3 model. The difference in the number of spines was calculated for different numbers of skipped planes for pure SIREN predictions (gray), denoised SIREN predictions (blue), and linear interpolation (green). Results were normalized with respect to the number of spines in the original image stack. **d**, Top: Visualization, how planes are selected. Gray planes are used for training and blue planes get predicted and therefore are skipped during training. The predicted planes were evaluated by comparing them to the corresponding planes of the originally acquired stack, which were skipped during training. Bottom left: Original image slice with annotations (pink: dendrites, green: spines) from DeepD3 model. Bottom right: pure SIREN predictions and denoised SIREN predictions for different numbers of skipped planes with annotations (pink: dendrites, green: spines) from the DeepD3 model.

In the next step, we trained SIRENs to predict intermediate planes of the motion-corrected image stack. In the preprocessing, planes are selected for training and prediction. During training, the SIREN learns an implicit representation of the 3D stack using the x-, y-, and z-coordinates of the training planes as input values and the corresponding intensity values as ground truth. Subsequently, the network predicts the intensity values of a grid corresponding to the planes selected for the prediction. These predictions are then further improved by a denoiser network. Figure 3(d) visualizes the process of acquiring planes for training the SIRENs and predicting interplanes.

We evaluated the interplane prediction for the first 20 layers, i.e. 20 μ m, of ten different two-photon microscopy stacks. Figure 3(a) and (b) show the MSE and SSIM respectively for different numbers of skipped planes for the pure SIREN predictions (gray), the same predictions subsequently corrected by our denoiser network (blue), and interplanes reconstructed with linear interpolation. SSIM, MSE, and PSNR are nearly equal for one and two skipped planes when using SIRENs (green). With an increasing offset between the training planes, the SSIM drops from about 70% to 30%. The MSE increases from about 0.7 for pure SIRENs and one or two skipped planes to 4.4 with seven planes skipped. We found that by adding the denoiser network in the postprocessing step, all configurations consistently performed better (Fig. 3(a)-(c)). The difference in the SSIM between pure SIREN predictions and denoised SIREN predictions becomes smaller with an increasing offset. This is not the case for the MSE. Here, an increasing offset leads to an increased difference between pure SIREN predictions and denoised SIREN predictions. Linear interpolation performs better in all cases regarding MSE and SSIM. This is not surprising, since linear interpolation is a naive approach to sample between two adjacent and very similar planes. MSE and SSIM evaluate the general consistency between two images but do not necessarily show, how reliably details are reconstructed.

To evaluate the reconstruction of fine details, fundamental for the research conducted with the microscopic images, we evaluated how many dendritic spines of the original image stack can be recovered in the interplanes. Figure 3(c) shows an exemplary evaluation of the first twenty image planes of a single microscopy stack. We compared the number of spines of the planes from the original stack to the number of spines in the SIREN predicted stack, the denoised SIREN prediction, and the interplanes constructed by linear interpolation. The results were normalized to the number of spines in the original image stack. In the pure SIREN prediction, 76% of the spines could be reconstructed if only one layer is skipped. This value decreases down to 29% if seven layers are skipped. In combination with the denoiser network, an average of about 85% of the spines can be recovered for up to 3 omitted planes and even if 7 planes are skipped, more than 50% of spines can be recovered. Similarly to the reconstruction error, the percentage of recovered spines decreases with an increasing offset between the training planes. In comparison to spine reconstruction using linearly interpolated planes, the error of the SIREN predictions is lower or about equally low for up to 5 skipped planes. However, as the number of skipped planes increases, the error rate of SIREN predictions rises while that of linear interpolation decreases.

Figures 3(a)-(c) indicate that there is still an error between directly acquiring image planes and predicting intermediate planes with SIRENs. Acquiring only every n-th image plane and predicting the intermediate planes with SIRENs benefits from a shorter acquisition time and fewer negative effects such as photobleaching. The disadvantage is the lower quality of the predictions compared to originally acquired image stacks.

Figure 3(d) shows the DeepD3 segmentation results for the different numbers of omitted planes and compares the results of the pure SIREN predictions and denoised SIREN predictions exemplarily and qualitatively. Both are largely consistent with the originally acquired image if only one or two planes are skipped. With an increasing offset, the pure SIREN predictions introduce an increasing noise level and fewer spines can be identified. However, the SIREN predictions can be efficiently denoised and the spine identification can be improved, which can be evaluated by comparing the pure SIREN predictions with the denoised predictions.

### 3.3 Spine recovery

Interestingly, the spine reconstruction of the predicted image planes was about 20% to 40% with pure SIRENs. By mining our full pipeline, we found that using percentile normalizer in the preprocessing step (see section 2.2) and a custom denoiser network for postprocessing, as described in section 2.3, improve the spine recovery based on the segmentation of the DeepD3 framework.

We observed some interesting behaviors regarding the improvement of spine reconstruction by the denoiser network. As long as there is only a low or medium number of spines in one image plane, the spines lost in the SIREN reconstruction can be reliably recovered by the denoiser. But if the number of spines exceeds a specific level, the impact of the denoiser decreases and the number of spines, which DeepD3 can segment, does not change or is even reduced in the denoised SIREN prediction compared to the pure SIREN prediction. With our data, image planes with more than about 40 spines had mainly an equal or worse spine reconstruction in the denoised prediction compared to the pure SIREN prediction. These effects occur if the processed images have a medium or high noise level with respect to the training data of the denoiser network.

**Fig. 4.**
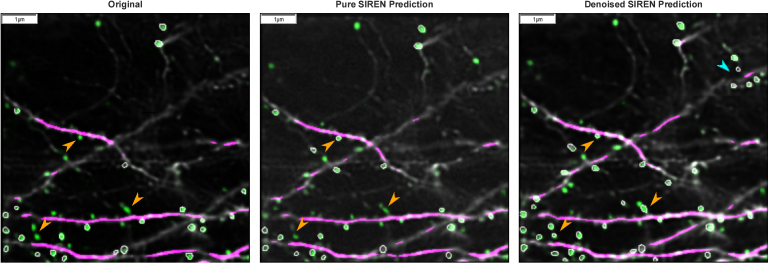
**Spine Recovery.** Comparison of the dendritic spine and dendrite segmentation by DeepD3 of the originally acquired image (left), the pure SIREN prediction (middle), and the denoised SIREN predictions (right). Green pixel intensities denote the probability of being a spine. Green pixel areas with white borders were annotated as a spine. Orange arrowheads point to pixel areas, which have a medium or low probability of being a spine and are therefore not annotated as a spine in the originally acquired image (and the pure SIREN prediction), but have an increased probability of being a spine and are therefore annotated as a spine in the denoised SIREN predictions. The blue arrowhead points to spines identified in the denoised SIREN predictions, which had zero probability of being a spine in the originally acquired image. The effect of a higher number of annotated spines in the denoised SIREN predictions than in the originally acquired image occurs if the pure SIREN prediction already has a low noise level, which is further reduced by the denoiser network and falls below the noise level of the originally acquired image. All reconstructed planes including the annotations from DeepD3 are shown in the video Visualization 1 in the supplementary material, comparing pure SIREN predictions, denoised SIREN predictions, and linear interpolation.

For images with a rather low noise level, we observed another interesting effect in the denoised SIREN predictions. The denoiser reduces noise in the SIREN predictions. If an input image has already only low noise, the SIREN prediction will also have only a low noise level. In these cases, the spine recovery of the pure SIREN prediction is already really good. We analyzed an image stack with 70 image planes, where we observed an average of 84% recovered spines with a standard deviation of 2.4 spines per image plane in the pure SIREN prediction. As this noise level is further reduced by the denoiser, the noise in the denoised SIREN prediction is lower than in the originally acquired image. In these images, more spines are identified by DeepD3 than in the input image and SIREN predictions, probably because structures are more clearly visible due to the lower noise level. In the denoised image stack, DeepD3 segmented about 50% more spines than in the originally acquired image stack. In most cases, areas, that already had a medium probability of being a spine, but were not annotated as a spine in the original image due to the user settings in the DeepD3 GUI had an increased probability of being a spine in the denoised image planes, and were therefore after the correction step segmented as spines. This results in an increased number of spines in the denoised image planes. This effect is shown in Fig. 4.

## 4. Discussion

Unsupervised motion correction is a novel application of implicit neural representations that avoids manual effort in selecting planes without motion artifacts and incorporates the maximum knowledge available through scanning repetitions. Our method is able to outperform a state-of-the-art CNN for motion correction. Additionally, our model is unsupervised, which means there is no need for additional data besides the acquired images of the stack that should be corrected. To train the baseline model a training dataset containing multiple image stacks is necessary.

However, to correct motion artifacts with SIRENs, a network has to be trained for each stack. To reduce the training time of the SIRENs, we gradually decreased the pixels of the training data, which led to a proportional decrease in the training time. Recent works show additional modifications, which lead to a further improved speed. For instance, [18] accelerate the training and inference speed of implicit neural representations by splitting the initial layers of the MLP to learn each input dimension independently. Subsequently, the last layers merge the intermediate features to generate the learned signal at each corresponding coordinate point. This approach yields a speedup of up to 2.92x while maintaining comparable accuracy levels to the baseline model.

Our interplane prediction approach was inspired by the ability of SIRENs to perform inpainting [9], which allows the reconstruction of missing pixels according to adjacent information. As implicit neural representations model the underlying shape of a signal, analyzing this signal is not limited to the acquisition grid anymore, but signals can be analyzed at arbitrary positions. Therefore intermediate planes of an image stack can be predicted, which allows for higher offsets between acquisition planes and a finer resolution between the acquired planes. The predicted interplanes preserve important neuroanatomic structures as long as the offset between the initially acquired planes is not too large. A potential reason for this limitation is, that some structures are smaller than the offset between the training planes, and the information about these structures gets lost.

When comparing our approach to linear interpolation between image planes, we found that linear interpolation performs better than our approach with respect to MSE and SSIM. We hypothesize that linear interpolation directly uses the originally acquired planes and adjacent planes contain highly repetitive information. But when comparing the number of spines in interpolated and SIREN reconstructed images, it is higher or almost equal in interpolated planes for up to 5 skipped planes. We believe that in the interpolated plane, spines are derived from information in both adjacent planes, resulting in the identification of numerous additional spines compared to the originally acquired planes. This effect decreases when more intermediate planes are predicted between two images since spines are more weighted to one or the other plane. Therefore we assume, that SIRENs are capable of assigning spines to specific planes, which linear interpolation can do only partially.

We counted the number of spines of different image stacks with the deep learning framework DeepD3 and observed some interesting effects. By correcting the pure SIREN predictions with a denoiser network, special behavior can be observed depending on the input characteristics. For image stacks with an average to high noise level and a medium number of spines, the denoised predictions turn out as intended. If the number of spines exceeds a specific level (about 40 spines in our case), an equal or lower number of spines can be identified in the denoised SIREN predictions compared to the originally acquired image and the SIREN prediction. Potentially this effect could occur because the denoiser network has problems differentiating between noise and many spines close to each other, such that some spines get removed. Another effect occurs if the noise level of the input data is rather low compared to the noise in the training data of the denoiser network. In this case, the probability of being a spine of some pixel areas increases in the denoised SIREN predictions. Therefore more spines are identified in the denoised SIREN predictions than in the originally acquired image and the pure SIREN prediction.

Dendritic spine quantification is, in general, an error-prone process with high inter-rater reliability (82.2 ± 6.4%) [17]. We have determined, that the number of spines quantified in our final predictions depends on the noise level of the training data of our denoiser network, which then influences the number of identified spines. Therefore we come to the assumption, that we can control the confidence in predicting a spine, by the training data we use for our denoiser network. We assume that training a denoiser network with data impacted by a rather high noise level as input data and data with a low noise level as ground truth leads to predictions with lower noise than in the ground truth for input images with an already low noise level. Future work should evaluate if this could potentially make dendritic spine quantification easier than in the originally acquired microscopic image stacks, by not only generating an implicit reconstruction but further improving its quality.

In our work, we utilized the capability of SIRENs to decipher stable data from motion-affected data and applied it to predict coordinate values independently of the original acquisition grid of microscopic images. It is worth noting that these characteristics extend beyond the scope of our work and can be effectively employed across a diverse field of data types that contend with motion artifacts or are challenging to acquire. This broader applicability is particularly relevant for large-scale data, where manual identification of artifacts is not feasible or data acquisition processes are characterized by significant time and financial investments, as well as associated risks.

## 5. Conclusion

In this study, we have shown that SIRENs are a powerful architecture in retaining and processing microscopy image stacks. Especially in combination with carefully set preprocessing steps and deep neural networks that denoise the SIREN predictions while preserving the relevant neuroanatomic structures in the postprocessing, we can show with relevant neuroscientific metrics, such as dendritic spine recovery, that implicit neural representations will play a relevant part in the future of biomedical image processing. We are able to create motion-corrected microscopic image stacks from multiple acquisitions of the same stack with unknown components of motion. Our method is superior to the common approach of averaging the acquired stacks, where motion artifacts mathematically still contribute to the resulting data. Our method can also replace the cumbersome process of manually removing motion-afflicted data. Additionally, we are able to predict intermediate planes that reliably reconstruct relevant neuroanatomic structures. With our methods planes can be acquired with a higher offset, which accelerates the acquisition and reduces negative effects like photobleaching. Additionally, the implicit neural representations by SIRENs allow for analyzing the sample at arbitrary positions.

## Data Availability

Data underlying the results presented in this paper are not publicly available at this time but may be obtained from the authors upon reasonable request.
